# Retinal Vascular Pathology in MCI and AD: Links to Cerebral Amyloid Angiopathy and Cognition

**DOI:** 10.1002/alz70855_100907

**Published:** 2025-12-23

**Authors:** Maya Koronyo‐Hamaoui, Jonah Doustar, Yosef Koronyo, Dieu‐Trang Fuchs, Altan Rentsendorj, Julie A Schneider, Lon S. S Schneider, Keith L Black, Roxana O. O Carare, Haoshen Shi, Oana M Dumitrascu

**Affiliations:** ^1^ Department of Neurosurgery, Maxine Dunitz Neurosurgical Research Institute, Cedars‐Sinai Medical Center, Los Angeles, CA, USA; ^2^ Department of Neurology, Cedars‐Sinai Medical Center, Los Angeles, CA, USA; ^3^ Cedars‐Sinai Medical Center, Los Angeles, CA, USA; ^4^ Department of Biomedical Sciences, Division of Applied Cell Biology and Physiology, Cedars‐Sinai Medical Center, Los Angeles, CA, USA; ^5^ Department of Neurology and Pathology, Rush University Medical Center, Chicago, IL, USA; ^6^ Rush Alzheimer's Disease Center, Rush University Medical Center, Chicago, IL, USA; ^7^ Alzheimer's Disease Research Center, Keck School of Medicine, University of Southern California, Los Angeles, CA, USA; ^8^ Faculty of Medicine, University of Southampton, Southampton, United Kingdom; ^9^ Neurosurgery Department, Cedars‐Sinai Medical Center, Los Angeles, CA, USA; ^10^ Mayo Clinic College of Medicine and Science, Scottsdale, AZ, USA

## Abstract

**Background:**

The interplay between vascular dysfunction and brain amyloidosis in driving Alzheimer's disease (AD) pathogenesis and cognitive decline is increasingly recognized. The retina, sharing an embryonic origin with the brain, offers an accessible CNS organ for high‐resolution, noninvasive imaging of AD‐related vascular pathology. However, the exact nature of retinal vascular damage in AD—especially in early disease stages—and its relationship with cerebral amyloid angiopathy (CAA) and cognitive impairment remain understudied.

**Method:**

We examined vascular abnormalities in retinas of MCI and AD via histopathology and in‐vivo imaging studies. Postmortem retinas from 53–62 individuals (CN: 22; MCI/AD: 31–40) underwent immunohistochemistry for pericyte integrity, vascular Aβ42/40, and endothelial TJ proteins, with dysregulated proteins quantified by mass spectrometry in 12 additional AD/CN cases. Retinal curcumin‐amyloid imaging by scanning‐laser ophthalmoscope in 28–34 live participants (CN[MOCA>26]: 8–15; MCI/AD[MOCA≤26]: 13–19) assessed peri‐arteriolar/venular amyloid plaques (APs). Perivascular AP burden was correlated with MRI measures (hippocampal volume, WMH lesions) and cognitive/neuropsychiatric parameters.

**Result:**

Retinal pericyte loss, reflected by PDGFRβ deficiency and apoptosis, was evident in MCI and AD patients and correlated with vascular Aβ42/40 accumulation and CAA severity (r=0.68–0.84; *p* <0.01–0.0001). Retinal endothelial ZO‐1 and claudin‐5 levels were significantly reduced, correlating with arteriolar Aβ40 deposition, suggesting impaired retinal Aβ clearance. Retinal capillary claudin‐5 and arteriolar Aβ40 strongly associated with CAA (r=0.75–0.77, *p* <0.0001), while ZO‐1 moderately correlated with brain amyloidosis/tauopathy, CAA, and cognitive decline (r=0.43–0.57, *p* <0.01–0.001). Both histological and in‐vivo imaging studies revealed greater Aβ deposition in retinal arterioles, particularly in secondary and tertiary branches, compared to venules or capillaries (*p* <0.01–0.0001). Retinal perivascular APs in secondary/tertiary branches imaged in living patients correlated with cognitive impairment (global CDR, MOCA), hippocampal atrophy, and WMH lesions (r=0.51–0.66, *p* <0.05–0.001). Perivascular APs in the secondary (β=0.397, *p* = 0.033) and tertiary (β=0.756, *p* = 0.017) arteriolar branches showed significant interactions with hippocampal volumetry for visual memory.

**Conclusion:**

Retinal arteriolar Aβ accumulation, pericyte loss, and TJ dysfunction contribute to early AD vascular pathology. If validated in larger cohorts, these findings support retinal vascular biomarkers as tools for predicting cognitive decline, monitoring AD progression, and assessing CAA severity.

